# Mortality associated with seizures in dementia

**DOI:** 10.1002/alz.088617

**Published:** 2025-01-09

**Authors:** Ifrah Zawar

**Affiliations:** ^1^ University of Virginia, Charlottesville, VA USA

## Abstract

**Background:**

Seizures are a common co‐morbidity of dementia and are associated with accelerated cognitive decline. However, the impact of recurrent versus remote seizures on mortality outcomes in people with dementia (PWD) has not been studied. The purpose of our study is to fill this knowledge gap.

**Method:**

This longitudinal, multicenter study is based on participants recruited from 39 Alzheimer’s disease Centers in the US from 9/2005 –12/2021. The effect of recurrent or active seizures (over the preceding 12 months and/or requiring active treatment), remote seizures (history of seizures but no seizures over the preceding 12 months) and no history of seizures (controls) were assessed on mortality outcomes in people with dementia.

**Result:**

Among 26,598 cognitively impaired, 664 had recurrent seizures and 567 had remote seizures. Early onset of cognitive decline (p<0.001), dominant AD mutation (p = 0.002), Parkinson’s disease (PD, p = 0.055), traumatic brain injury (TBI, p<0.001), active depression (p<0.001) and stroke or TIA were associated with active or remote seizures. Patients with active seizures performed worse on Clinical dementia rating (P<0.001), and functional assessment scale (p<0.001) compared to those without seizures.

Analysis of longitudinal mortality data showed that a higher proportion of active seizure patients had died compared to remote seizures and controls (35.86 vs 28.62 vs 19.11% respectively, p < 0.0001), and they were younger at death (74.88 vs 76.53 vs 79.81 years, p < 0.001). To examine mortality further, a Cox regression model was conducted to study age at death and seizure status. We adjusted the model for sex, race, ethnicity, age, severity of dementia, hypertension, diabetes, stroke, TIA, TBI, PD, depression, education, alcohol use and dominant AD mutation. Despite adjustment, patients with active seizures were at a higher risk of dying at a younger age (adjusted hazard ratio: aHR: 1.79, CI: 1.52‐2.10, p<0.001) but those with remote seizures were not (aHR: 1.05, CI:0.90‐1.24, p0.52).

**Conclusion:**

Our research findings suggest that PWD with active or recurrent seizures have higher mortality rates at a younger age compared to PWD with remote or no seizures. Our findings highlight the need for more aggressive control of seizures in PWD.

